# Chemical pleurodesis – a review of mechanisms involved in pleural space obliteration

**DOI:** 10.1186/s12931-019-1204-x

**Published:** 2019-11-07

**Authors:** Michal Mierzejewski, Piotr Korczynski, Rafal Krenke, Julius P. Janssen

**Affiliations:** 1 Department of Internal Medicine, Pulmonary Diseases & Allergy, Medical University of Warsaw, Warsaw, Poland; 2 Department of Pulmonary Diseases, Canisius-Wilhelmina Hospital, Nijmegen, The Netherlands

**Keywords:** Pleural effusion, Pleural fluid, Spontaneous pneumothorax, Pleurodesis, Sclerosing agents, Talc

## Abstract

Chemical pleurodesis is a therapeutic procedure applied to create the symphysis between the parietal and visceral pleura by intrapleural administration of various chemical agents (e.g. talk, tetracycline, iodopovidone, etc.). The two major clinical conditions treated with chemical pleurodesis are recurrent pleural effusion (PE) and recurrent spontaneous pneumothorax. Although the history of chemical pleurodesis began over a century ago, detailed data on the mechanisms of action of sclerosing agents are highly incomplete. The following article aims to present the state of knowledge on this subject.

It is believed that mesothelial cells are the main structural axis of pleurodesis. In response to sclerosing agents they secrete a variety of mediators including chemokines such as interleukin 8 (IL-8) and monocyte chemoattractant protein (MCP-1), as well as growth factors - vascular endothelial growth factor (VEGF), platelet-derived growth factor (PDGF), basic fibroblast growth factor (bFGF) and transforming growth factor- β (TGF-β). Numerous data suggest that intact mesothelial cells and the above cytokines play a crucial role in the initiation and maintenance of different pathways of pleural inflammation and pleural space obliteration.

It seems that the process of pleurodesis is largely nonspecific to the sclerosant and involves the same ultimate pathways including activation of pleural cells, coagulation cascade, fibrin chain formation, fibroblast proliferation and production of collagen and extracellular matrix components. Of these processes, the coagulation cascade with decreased fibrinolytic activity and increased fibrinogenesis probably plays a pivotal role, at least during the early response to sclerosant administration.

A better understanding of various pathways involved in pleurodesis may be a prerequisite for more effective and safe use of various sclerosants and for the development of new, perhaps more personalized therapeutic approaches.

## Background and clinical aspects of pleurodesis

The term ‘pleurodesis’ comes from the Greek words *pleurá* (pleura) and *desmos* (bond) and refers to a procedure undertaken to create the symphysis between the parietal and visceral pleura in order to eliminate the pleural space. The procedure is applied to prevent the recurrence of spontaneous pneumothorax or pleural effusion.

Two major methods can be used to achieve pleurodesis: 1) direct injury to the pleura with mechanical or physical methods (e.g. mechanical abrasion, laser or argon beam coagulation) during video-assisted thoracoscopic surgery (VATS) or 2) intrapleural administration of various agents (e.g. talk, bleomycin, tetracycline, iodopovidone, *Corynebacterium parvum*) that induce formation of pleural adhesions. As the vast majority of these agents are chemicals, the term “chemical pleurodesis” is commonly used. Since chemical sclerosants can be administered to the pleural space by a pleural catheter or single point entry medical thoracoscopy, chemical pleurodesis is a less demanding and less invasive procedure than VATS and is therefore more commonly used.

The two major clinical conditions that can be treated with chemical pleurodesis are recurrent pleural effusion and recurrent spontaneous pneumothorax. The concept of applying chemical pleurodesis in these two entities is slightly different. In patients with recurrent pleural effusion, pleurodesis is a symptomatic treatment that prevents dyspnea related to relatively rapid reaccumulation of pleural fluid after its earlier withdrawal. Thus, the beneficial effect of pleurodesis in patients with pleural effusion can be observed within weeks or months after the procedure. In recurrent spontaneous pneumothorax, pleurodesis has a more delayed effect (months, years), as it is not applied to treat the current pneumothorax episode, but it is aimed to prevent future pneumothorax recurrences.

Symptomatic treatment of recurrent pleural effusion is by far the most common indication for pleurodesis. Though it mainly refers to malignant pleural effusion (MPE), chemical pleurodesis can also be applied in patients with other underlying diseases. In developed countries, MPE is probably the third leading cause of pleural effusion, with approximately 200,000 new cases diagnosed each year in the United States [1]. Almost 75% of all MPEs are associated with three common malignancies: lung cancer, breast cancer and lymphoma. According to literature data as many as 30% of lung cancer patients and 7–11% of breast cancer patients develop pleural effusion [2]. Furthermore, it is estimated that malignant pleural effusion may occur in nearly 50% of all patients with metastatic cancers [3]. Since MPE usually reflects advanced (metastatic) disease, curative treatment is only occasionally possible and effective. Thus, although chemical pleurodesis is a palliative treatment that should be applied only in patients who cannot be offered effective causative treatment, or in those in whom causal treatment was found to be ineffective, the number of patients who require symptomatic treatment due to dyspnea related to MPE is substantial. The number of pleurodeses performed in the United States approximates 100,000 per year [4]. The treatment strategy in patients with symptomatic MPE is complex but pleural interventions play a pivotal role. The choice of therapeutic procedure should be based on several determinants, including life expectancy and patient preferences [5]. In terms of prognosis in patients with MPE the first validated prognostic scores to predict survival in patients with MPE has been developed in recent years, and they seems to be very helpful in selecting patients who might benefit from pleurodesis [6, 7]. Large volume therapeutic thoracentesis may bring an immediate therapeutic effect but it does not prevent pleural fluid reaccumulation. Therefore, repeated therapeutic thoracenteses might be recommended only for patients with a slow rate of pleural fluid reaccumulation (> 1 month), patients with a short-expected survival time (< 3 months) [8], and some patients who will undergo chemotherapy or targeted therapy which is expected to be effective against reaccumulation of pleural effusion, like in small cell lung cancer, lymphoma, or adenocarcinoma with EGFR mutation. In the remaining patients, procedures that reduce the future risk of pleural effusion recurrence should be considered, with chemical pleurodesis being the major therapeutic option. It must be emphasized, however, that not all patients with recurrent malignant pleural effusion are suitable candidates for chemical pleurodesis. The two major contraindications to the procedure are an unexpandable lung (“trapped lung”) and inability to remove pleural fluid, e.g. due to multiple loculations. This is because the formation of effective pleural adhesions after administration of sclerosing agent requires a close and direct contact between the parietal and visceral pleura. Thus, an effective withdrawal of pleural effusion and the ability of the lung to reexpand are important prerequisites for efficient pleurodesis. The overall effectiveness of chemical pleurodesis in prevention of reaccumulation of MPE ranges between 41.3 and 100% and depends on different factors, including cancer type, extension of pleural involvement and the sclerosant used for pleurodesis [9–11]. Another increasingly popular therapeutic option that might also be offered to patients with unexpandable lung is chest tube insertion to permanently drain the pleural fluid (indwelling or tunneled pleural catheter, IPC). Pleuro-peritoneal [12] or pleuro-venal [13, 14] shunts are hardly ever used anymore, because of the invasive character of the procedure and the high complication rate. With IPC, symptomatic relief can be achieved in 70–94.2% patients with MPE [15, 16]. Moreover, it has been shown that drainage of the pleural cavity may lead to spontaneous pleurodesis [17].

Recurrent spontaneous pneumothorax is the second most common indication for chemical pleurodesis. Data on the incidence of spontaneous pneumothorax are rather scarce. The results of one recent study that used an English national dataset showed the overall annual incidence for both primary and secondary spontaneous pneumothorax of 14.1 per 100,000 population [18]. This number seems to be consistent with the data from earlier studies [19–21]. Most authors agree that pleurodesis should be offered to patients with the first recurrence (i.e. the second episode) of spontaneous pneumothorax. However, the definitive treatment aimed to prevent recurrence of pneumothorax is not limited to chemical pleurodesis, but also includes other therapeutic options [22, 23]. Bearing in mind the above considerations, it is easy to understand that the number of patients with spontaneous pneumothorax who are treated with chemical pleurodesis might be lower than the respective number of patients with pleural effusion. Nevertheless, chemical pleurodesis is a widely used, cost-effective method preventing recurrence of primary spontaneous pneumothorax. The effectiveness of chemical and mechanical pleurodesis in this group of patients is high and estimated to reach 90–99% [22, 23].

Available data indicate that chemical pleurodesis is an important treatment option for both patients with pleural effusion and spontaneous pneumothorax. Hence, the importance of understanding the mechanisms of action of sclerosing agents and further studies on new effective and safe sclerosants.

## History, agents and basic mechanisms involved in chemical pleurodesis

The history of pleurodesis dates back to 1901, when a Swiss surgeon, Lucius Spengler performed the first procedure intended to produce adhesion between the visceral and parietal pleura. However, the results of intrapleural instillation of a hypertonic glucose solution were unsatisfactory. Thus, as early as in 1906, the same author suggested the use of 0.5% silver nitrate solution instead of hypertonic glucose [24, 25]. In 1935, Norman Bethune proposed intrapleural application of iodized talc to create pleural adhesions in patients with bronchiectasis prior to lobectomy [26]. The use of talc for palliative treatment of MPE was first described by John Chambers in 1958 [27]. To date, it remains one of the most effective and widely used sclerosing agents [28].

In the over a hundred-year history of pleurodesis, a variety of methods have been proposed and tested to achieve effective pleural symphysis. These include both mechanical abrasion (first performed by an American surgeon, Edward Delos Churchill in 1941 [29]) and different chemical sclerosants, i.e. antibiotics (tetracycline [30], erythromycin [31], minocycline [32], doxycycline [33]), antiseptics (silver nitrate [34], iodopovidone [35]), cytostatic agents (mitomycin C [36], bleomycin [37], cytarabine [38], doxorubicin [39], etoposide [40], mitoxantrone [41], nitrogen mustard [42]), radioactive colloidal gold [43], quinacrine [44], transforming growth factor β (TGF-β) [45], autologic blood [46], lipoteichoic acid-T [47] or even bacteria (*Corynebacterium parvum* [48]*, Streptococcus pyogenes* A3 (OK-432) [39]. The search for the ideal sclerosing agent is still ongoing.

Currently, it seems that formation of fibrin adhesions and fibrosis are necessary processes to create a permanent symphysis between the visceral and parietal pleura [49]. Although different pathways can lead to formation of pleural adhesions, inflammation is the most important and common mechanism involved in pleurodesis. This mechanism includes the production and release of cytokines as well as adhesion molecules leading to activation of the coagulation cascade and an imbalance between fibrinolysis and fibrinogenesis in favor of the latter. A further consequence of these processes is fibroblast recruitment and proliferation. Nearly all sclerosants used for pleurodesis act as local irritants [50] that induce local inflammation eventually resulting in creation of pleural adhesions. In fact, the involvement of inflammation in the formation of pleural adhesions is disadvantageous because it is associated with side effects, including pain. However, to date there is no easily available and efficient sclerosant that shows strong pro-adhesive but no pro-inflammatory activity. It is believed that the ideal pleural sclerosant should produce durable adhesions with as little as possible or even no inflammation.

Although used for many years, detailed data on the mechanisms of action of various sclerosing agents are highly incomplete. This refers, for example, to iodopovidone which still seems to be an interesting and promising sclerosant [51]. The pro-inflammatory effect of this agent was tested only in animal models [52, 53]. Most of the studies performed to date focused on the agents which gained most popularity in different periods throughout the history of pleurodesis, e.g. talc, doxycycline and silver nitrate.

## Inflammation

Almost all sclerosing agents induce a nonspecific organizing fibrinous pleuritis, that leads to pleural fibrosis. Additionally, talc elicits a histiocytic and granulomatous foreign body reaction [54].

The significant role of the inflammatory process as a key pathway of pleurodesis has been demonstrated in animal studies which showed reduced efficacy of sclerosants when used together with nonsteroidal anti-inflammatory drugs (NSAIDs) [55]. However, a recent clinical study in humans showed that use of NSAIDs versus opiates resulted in non-inferior rates of pleurodesis [56]. Studies on animal models of talc and doxycycline pleurodesis demonstrated a significantly increased risk of pleurodesis failure during corticosteroid therapy [57, 58]. The same effect of corticosteroids was found for iodopovidone pleurodesis [52]. Interestingly, this phenomenon was not observed in the animal model when TGF-β was used as a sclerosing agent [59]. This raised hope for the future possibility to perform pleurodesis with selected cytokines with no side effects associated with inflammation and without a significant risk of pleurodesis failure in patients receiving anti-inflammatory therapies. However, there still have been no adequate clinical trials with TGF-β and therefore the use of this cytokine cannot be recommended for routine clinical practice.

### The role of mesothelial cells

**Fig. 1:**
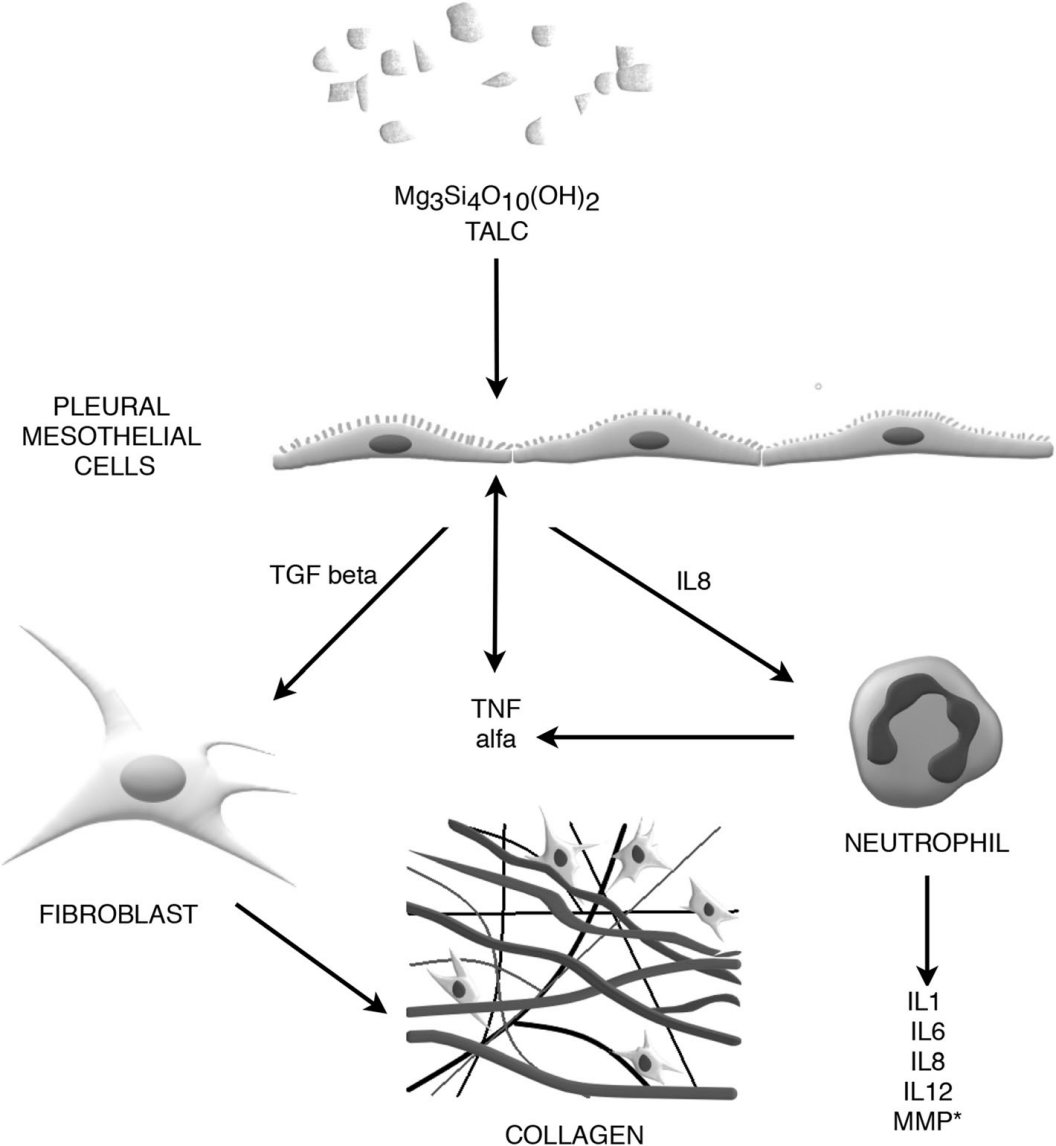
The role of inflammation in pleurodesis. In brief, inflammation is the most common mechanism involved in pleurodesis. Mesothelial cells which are directly exposed to the sclerosing agent initiate the chain of inflammatory reactions by secretion of a variety of mediators. These include the influx of neutrophils induced by interleukin 8 (IL-8) produced by mesothelial cells; see text for details. Solid lines represent stimulation; *- see also the role of matrix metalloproteinases (MMPs) in Fig. 3. Please, note that the figure is a simplified diagram summarizing the inflammatory mechanisms involved in pleurodesis. The actual mechanisms are probably far more complex

Originally, it was supposed that pleurodesis is closely related to massive damage of the surface of mesothelium caused by the sclerosing agent [60]. Meanwhile, more data were gathered pointing to the role of mesothelial cells as the major target for sclerosing agents and the key structure initiating the chain of inflammatory reactions. Thus, it is believed that mesothelial cells are the main structural axis of the inflammatory process. In response to sclerosing agents, mesothelial cells secrete variety of mediators which play a crucial role in different pathways of inflammation (Fig. 1). These include: chemokines, such as interleukin 8 (IL-8) and monocyte chemoattractant protein (MCP-1), growth factors - vascular endothelial growth factor (VEGF), platelet-derived growth factor (PDGF) and basic fibroblast growth factor (bFGF), transforming growth factor β (TGF-β) [61], and other mediators. Hence, the close contact between the sclerosing agent and a possibly large number of intact mesothelial cells seems to be crucial for effective pleurodesis. It is important to realize that although the mesothelial cells are the key player in the initiation of pleurodesis, the process is very complex and also involves other cells, including neutrophils, endothelial cells, fibroblasts, macrophages and cancer cells as well as various mediators.

**Interleukin 8** is a potent chemokine, which induces neutrophil influx into the pleural space [62]. Under normal conditions, IL-8 is produced by mesothelial cells and its production significantly increases in response to inflammatory stimuli [63]. Marchi et al. assessed the response of pleural mesothelial cells (PMCs) to talc in an experimental model and found that pleural fluid IL-8 concentration peaked at 6 h after intrapleural talc injection [64]. Thus, sclerosing agents may induce neutrophil influx to the pleural cavity, particularly during the first 24 h after administration [65]. The neutrophil count correlates with IL-8 concentration [66]. It has been demonstrated that neutrophil chemotactic activity in the pleural space is mainly related to IL-8, and it can be blocked by anti-IL8 antibodies [67]. Pleura migrating neutrophils produce and release a variety of other cytokines responsible for maintaining an already activated inflammatory pathway [50]. These include tumor necrosis factor α (TNF-α), IL-1α, IL-6, IL-1β and IL-12.

**TNF-α** is a proinflammatory cytokine derived from different sources, including PMCs. *In vitro,* it stimulates mesothelial cells to produce other cytokines e.g. IL-8 and VEGF [62]. In animal model, polyclonal anti-TNF-α Fab (antigen-binding fragment) decreased the effect of pleurodesis with talc. This observation emphasizes the role of TNF-α in talc induced pleurodesis. Interestingly, the same antibody did not affect pleurodesis with doxycycline [68].

**TGF-β** belongs to a family of growth factors that can modulate an inflammatory process. It acts as a chemoattractant for fibroblasts and shows both profibrotic and anti-inflammatory properties. Animal studies have shown that TGF-β stimulates fibroblasts and PMCs to synthesize collagen and induces pleurodesis but these effects are not associated with IL-8 release and an acute inflammatory response [69]. Injecting TGF-β into the rabbit pleural cavity increases the level of VEGF in the pleural fluid; in vitro studies have shown that TGF-β stimulates mesothelial cell VEGF production [45]. As to date TGF-β has been tested only in experimental animal models, its future use in clinical practice must be preceded by further research.

Mesothelial cells are also the source of other mediators that may be involved in formation of pleural symphysis. The role of these factors is discussed in the next sections of the paper.

The pivotal role of PMCs in pleurodesis is consistent with the hypothesis and observations on the relationship between the number of the mesothelial cells that can respond to a sclerosant and the efficacy of pleurodesis. This phenomenon is probably related to the quantitative characteristics of inflammatory reaction induced by sclerosant instillation. Although there has been no direct evidence on the relationship between the number of intact mesothelial cells and the efficacy of pleurodesis, several studies provided data that may be construed as an indirect confirmation of such a relationship. Rodriguez-Panadero et al. showed that tumor burden in the pleural space (resulting in the reduction of normal mesothelium) significantly correlated with low values of pleural fluid pH [70]. Further, the success rate of talc pleurodesis in MPE was significantly associated with pleural fluid pH (and possibly with the amount of normal mesothelium). Effective pleurodesis was achieved in 79% of patients with pleural fluid pH equal or higher than 7.30, in 40% of those with pH lower than 7.20, while failed in all patients with pH lower than 7.15. The results might indicate that an insufficient amount of unchanged PMCs, (in case of extensive pleural involvement by cancer) may result in ineffective pleurodesis. On the other hand, it must be admitted that pleural fluid pH in MPE is associated with the risk of lung entrapment and increased mortality. Also, it was suggested, that it is not only associated with impaired lung re-expansion, but also with inhibition of the biological processes leading to pleurodesis [71]. The role of the PMC count may be confirmed by the observation that pleurodesis in spontaneous pneumothorax which is characterized by a large number of intact mesothelial cells requires smaller doses of the sclerosing agents [50].

## Coagulation cascade

**Fig. 2:**
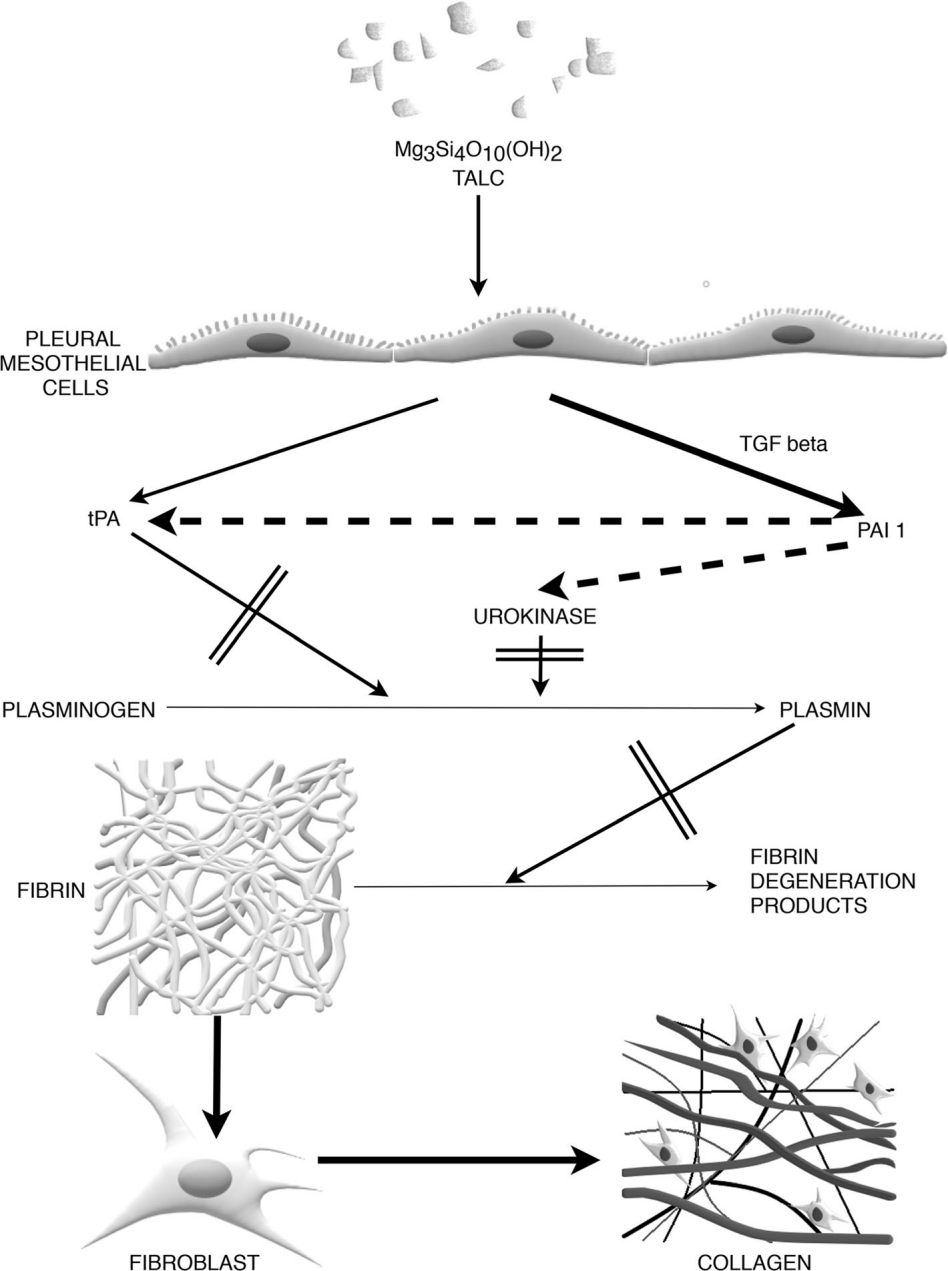
The role of coagulation in pleurodesis. In brief, under physiological conditions there is a balance between fibrinogenesis and fibrinolysis in the pleural space. This balance is determined by a sustainable release of a tissue plasminogen activator (tPA) and plasminogen activator inhibitor-1 (PAI 1) from pleural mesothelial cells. Intrapleural application of the sclerosing agents result in decreased activity of fibrinolysis and increased activity of intrapleural coagulation. These processes lead to the formation of fibrin mesh; see text for details. Solid lines represent stimulation, dashed lines represent inhibition; arrows with double strikethrough represent the mechanisms which significantly block the physiological pathways. This figure is a simplified diagram presenting the major pathways of fibrinolysis and fibrinogenesis during chemical pleurodesis. The actual mechanisms are probably more complex

Under normal physiological conditions there is a balance between fibrinogenesis and fibrinolysis in the pleural space. This depends on a sustainable release of a potent anticoagulant factor - tissue plasminogen activator (tPA) and plasminogen activator inhibitor-1 (PAI 1) that acts as a procoagulant. Both factors are secreted by mesothelial cells [72, 73]. The general effect of intrapleural application of the sclerosing agents can be seen as a decrease in activity of fibrinolysis and an increase in activity of intrapleural coagulation (Fig. 2).

Rodriguez-Panadero et al. demonstrated that successful pleurodesis was associated with significant reduction of the fibrinolytic activity expressed as pleural fluid D-dimer concentration 1 day after the administration of powdered talc (*p* < 0.001). Interestingly, pleural D-dimer level did not decrease in patients with treatment failure [74]. On the contrary, a significant increase in fibrinolysis was observed 3 h after intrapleural talc administration in those patients. This phenomenon was reported only after talc pleurodesis and it is not known whether other sclerosants produce the same effect.

The plasminogen activator inhibitor (PAI) pathway is another important mechanism evaluated in the context of pleurodesis. PAI reduces fibrinolytic activity in the pleural cavity by inhibiting the action of urokinase and tPA which, in turn, convert plasminogen into plasmin. PAI production by mesothelial cells is strongly stimulated by TGF-β [72].

Karandashova et al. [75] used a rabbit model with overexpression of human PAI-1 in the pleural mesothelium to study the mechanisms of tetracycline pleurodesis. The target gene was delivered by recombinant adenovirus and followed by tetracycline induced pleural injury. The PAI-1 overexpression itself was not a cause of pleural injury, effusion or fibrosis but significantly enhanced pleural injury, adhesion formation and density (number of adhesions) induced by tetracycline.

Tucker et al. [76] investigated pleural injury induced with carbon black/bleomycin in animal models including wild-type C57Bl/6j mice, PAI-1^−/−^ mice and PAI-1 transgenic mice overexpressing human PAI-1 (PAI-1^Tg^). In this study, fibrinous strands in the pleural cavity were found only in animals overexpressing PAI-1. All groups were positive for pleural fibrin antigen at 14 days after injury, however PAI-1^TG^ mice were found to have a significantly higher fibrin thickness in comparison to other groups. Interestingly, PAI-1^−/−^ mice exhibited significantly greater pleural thickening and more severe injury than the two other groups.

In the human study by Agrenius et al., PAI-1 activity was investigated in 10 patients undergoing pleurodesis with quinacrine (antimalarial drug) [77]. Pleural fluid PAI-1 level before pleurodesis was 21.7 +/− 12.0 (mean +/− SD) AU/ml and increased to 86.9 +/− 25.9 AU/ml 6 h after drug instillation. Furthermore, high D-dimer concentrations before treatment (62.7 +/− 25.5 mcg/ml) rapidly decreased 6 h after the procedure (12.2 +/− 7.9 mcg/ml). This observation confirms that fibrinolytic activity decreases and coagulation activity increases after intrapleural drug instillation. The limitation of the study was the lack of a control group.

The use of control groups in the studies on pleurodesis seems to be a relevant issue. This is because the possible changes in intrapleural fibrinolytic activity may be non-specific and may not be related to the sclerosing agent. Rodriguez-Panadero et al. [74] assessed PAI-1 activity in 75 patients who underwent thoracoscopy with or without talc instillation. Increased PAI-1 activity occurred not only after intrapleural talc administration but also after thoracoscopy and pleural drainage without pleurodesis. The reaction was similarly strong in both groups. Therefore, it appears that increased PAI-1 activity might be a non-specific phenomenon unrelated, or at least weakly related to the mechanism of action of the sclerosing agents.

The phenomenon of pro- and anticoagulation imbalance after intrapleural administration of chemical sclerosants is probably dependent not only on direct changes in the activity of coagulation cascade factors but also influenced by various cytokines and C-reactive protein which are produced by the mesothelium, endothelium, macrophages and inflammatory cells. Although the mechanisms are not fully understood, it seems that there is a close relationship between the local inflammation initiated by sclerosants and activation of coagulation cascade [78].

There is no convincing evidence on systemic activation of coagulation mechanisms in patients undergoing pleurodesis [79]. However, some cases of massive pulmonary embolism associated with pleurodesis have been observed [80]. The relationship between pleurodesis and activation of systemic coagulation was evaluated by Montes-Worboys et al. in 231 patients with MPE submitted to thoracoscopic talc poudrage. Early fatal outcome occurred in 17 patients and thrombotic events were observed in six of these patients. Sudden death occurred in 4 patients, and in all of them acute pulmonary embolism was suspected. It was confirmed in the only patient who underwent a post-mortem examination. Based on the above study, it cannot be excluded that the activation of plasma coagulation factors by inflammatory cytokines (e.g. IL-8) may be responsible for side effects such as systemic thromboembolic events, which may even result in early death of patients undergoing chemical pleurodesis. Higher IL-8 levels were observed in patients with early mortality after pleurodesis, but the difference was not statistically significant [80].

Irrespective to the mechanisms involved in formation of early fibrin bonds between the visceral and parietal pleura, it seems that the organization of fibrin mesh - the final product of coagulation cascade - is an indispensable condition for recruitment and subsequent proliferation of fibroblasts. Thus, the phenomenon is a prerequisite for the formation of more permanent adhesions. Impairment of these processes is strongly associated with treatment failure.

## Fibrogenesis and fibrolysis

Fibrogenesis and fibrolysis are processes involved in the regulation of fibrosis and extracellular matrix composition. Fibrogenesis refers to the formation and proliferation of fibers or fibrous tissue associated with wound healing, regeneration and preventing tissues from breaking down due to inflammation, necrosis, and release of lysines [81, 82].

**Fig. 3:**
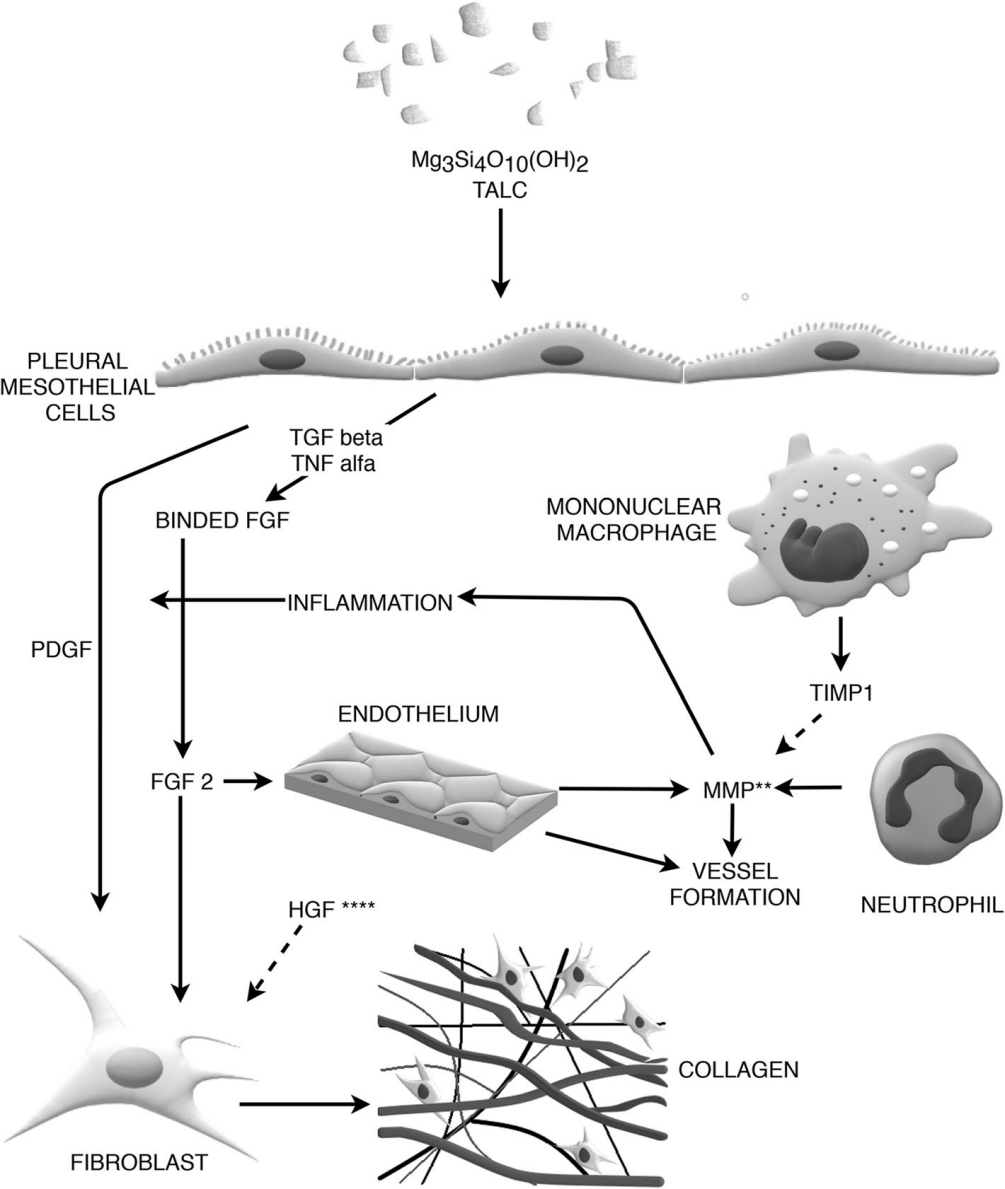
The role of fibrogensis and fibrolysis in pleurodesis. In brief, fibrogenesis occurs in the late stage of pleural symphysis formation and involves the recruitment and proliferation of fibroblasts producing collagen and extracellular matrix components in order to replace delicate fibrin pleural adhesions (see also Fig. 2) by stronger, dense collagen fibers; see text for details. Solid lines represent stimulation, dashed lines represent inhibition; **- see also role of matrix metalloproteinases (MMPs) in Fig. 1; *** - there have been no studies either on the effect of pleurodesis on HGF activity or potentially profibrogenic effects of anti-HGF antibodies in the pleural cavity. It can be hypothesized that inhibition of HGF might enhance pleural fibrogenesis initiated by the sclerosing agent. This figure is a simplified diagram, while the actual mechanisms are probably more complex

During pleurodesis, fibrogenesis occurs in the late stage of pleural symphysis formation and involves the recruitment and proliferation of fibroblasts producing collagen and extracellular matrix components. This leads to replacement of newly developed and delicate fibrin pleural adhesions formed as an early result of disbalance between coagulation and fibrinolysis (see above) by stronger bond of the dense collagen fibers. The process was elegantly showed by Hurewitz et al. in the study of the rabbit pleurae after the doxycycline pleurodesis [83]. Eventually (and ideally), the process results in the complete obliteration of the pleural cavity. Numerous growth factors affecting fibroblast function have been detected in the pleural cavity of patients treated with pleurodesis. These include: PDGF, bFGF, hepatocyte growth factor (HGF) and TGF-β (Fig. 3).

PDGF is synthesized by a variety of cell types, however, the major pleural source of PDGF are probably PMCs. In cell models unrelated to pleurodesis, its production was stimulated by low oxygen tension, thrombin, and other cytokines, e.g. IL-1 and IL–6 [84, 85]. PDGF plays an important role in fibroblast division by skipping the G1 checkpoints in the cell cycle. It shows pleiotropic activity, including chemotaxis, proliferation, and acceleration of extracellular matrix and collagen formation [86]. All the above effects might be important for efficient pleurodesis.

Basic fibroblast growth factor, also known as fibroblast growth factor 2 (FGF-2) is a member of fibroblast growth factor family which may play a considerable role in pleurodesis. It is produced by normal, but also by malignant mesothelial cells. Under physiological conditions, it is binded by proteoglycans, and may be released upon degradation of extracellular matrix by inflammatory cells [49]. In cell models unrelated to pleurodesis, its synthesis was stimulated by TNF-α, TGF-β and stem cell factor [87]. It seems that the main biological effect of bFGF is stimulation of the new blood vessel formation. Basic fibroblast growth factor also accelerates fibroblast proliferation and migration. However, the mechanism which is involved in regulation of fibroblast migration by bFGF is still unclear [88]. Some data from human studies suggesting that bFGF is important for efficient pleurodesis have been published. Antony et al. showed that patients who underwent successful pleurodesis had significantly higher pleural fluid bFGF levels than patients in whom pleurodesis had failed [49]. The authors hypothesized that lower pleural fluid bFGF levels in patients with extensive malignant involvement of the pleura are associated with a lower number of normal PMCs that can respond to sclerosing agent. Basic fibroblast growth factor production and release is probably an important component of this response. If this is true, higher pleural fluid levels of bFGF can be achieved in patients with less advanced pleural malignancies as more PMCs can be exposed to the sclerosing agent. This may result in a higher successful rate of pleurodesis in those patients.

Hepatocyte growth factor (HGF) has been initially identified as a mitogen for hepatocytes. Further studies showed that its activity is not limited to the liver, but it is also a potent mitogen, morphogen (a compound that controls tissue architecture) and stimulator of cell mobility in other organs including lungs [89]. The pleural effects of this cytokine were investigated in pleural fibrosis (fibrothorax) complicating pleural empyema [90]. It was demonstrated that HGF can cause opposite effects to those elicited by TGF-β and bFGF. Namely, it prevented adhesion formation and promoted the regeneration of the mesothelium cell layer. Several studies showed that anti-HGF antibodies induce fibrosis by neutralizing endogenous HGF. This phenomenon has been observed in animal models in the kidneys [91], liver [92] and lung diseases [93]. To our knowledge, there have been no studies either on the effect of pleurodesis on HGF activity or potentially profibrogenic effects of anti-HGF antibodies in the pleural cavity. However, it can be hypothesized that inhibition of HGF might enhance pleural fibrogenesis initiated by the sclerosing agent. This hypothesis should be tested in future studies. When discussing HGF in the context of MPE, it should be mentioned that one Japanese study showed an anti-tumor effect of anti-HGF and NK4 (a HGF fragment - competitive inhibitor of HGF-Met receptor) on malignant pleural mesothelioma [94].

Metalloproteinases (MMPs) and their inhibitors are yet another proteins involved in fibrogenesis and fibrolysis. MMPs are proteolytic enzymes, which are constitutively present in the pleura. They are secreted by mesothelial cells, fibroblasts, granulocytes and endothelial cells. Endogenous MMP inhibitors are a family of glycoproteins that is referred to as tissue inhibitors of metalloproteinases (TIMP). In the pleural cavity, TIMP-1 is secreted by mononuclear phagocytes [72]. Traditionally the role of MMPs and TIMPs was limited to remodeling of matrix components. Recent data suggest that their role is far more complex and includes intracellular and extracellular signaling [95]. Besides their enzymatic properties, the physiological functions of MMPs include wound healing, angiogenesis, organ morphogenesis and modulation of inflammatory processes. MMP9 activates IL-8 and inactivates IL-1β and is rapidly expressed as an early response to injury, persists during healing and declines upon re-epithelialization [96].

The metalloproteinase pathways in pleurodesis seem particularly intriguing because of their role in the development and progression of malignancies. MMPs are frequently overexpressed in malignant tumors and play a role in in situ tumor growth and spread. Moreover, some studies showed the role of MMPs in enhancement of inflammatory process [96]. However, despite the growing knowledge on the biology of MMPs, the understanding of their role in the pathogenesis of pleural effusion and pleurodesis is still limited.

Eickelberg et al. analyzed pleural effusions from 88 patients and suggested that interstitial collagenase (MMP-1), gelatinase-A (MMP-2), and TIMP-1 which are constitutively expressed in the lung may also play a role in homeostasis of the pleural space [97]. In a more recent study by Teixeira et al. that included 114 patients with various causes of pleural effusion, pleural fluid concentration of MMP1, MMP2, MMP8, MMP9 and TIMP correlated with other inflammatory mediators. Interestingly, there was a significant correlation between the level of MMP9 and TGF-β1 [98].

D’Agostino et al. demonstrated that talc pleurodesis was associated with the inhibition of MMP1 and TIMP1 synthesis in the pleural cavity. The effect of pleurodesis on MMP9 level varied between patients. In 4 patients, the level increased significantly a few days after pleurodesis, in 5 patients MMP9 levels were initially high and lowered after treatment. Although in all patients pleurodesis was successful, the authors did not discuss the potential relationship between MMPs/TIMPs changes and formation of pleural symphysis [99].

Hurewitz et al. showed that tetracycline and doxycycline inhibit the bioactivity of MMP2 when added to pleural fluid and in high concentrations reduce MMP2 synthesis in the fibrosarcoma cell culture [100]. This inhibitory effect was also observed in lower, subantimicrobal doses, but not in the context of pleurodesis [101]. The inhibition of MMP activity seems to be an attractive target for intrapleural therapy of MPE. Macaulay et al. showed a significant reduction in the number of recuired thoracenteses and better dyspnea scores in a group of 18 patients with MPE treated with intrapleural batimastat which is an anticancer drug with MMP inhibitor activity [102].

## Angiogenesis and angiostasis

**Fig. 4:**
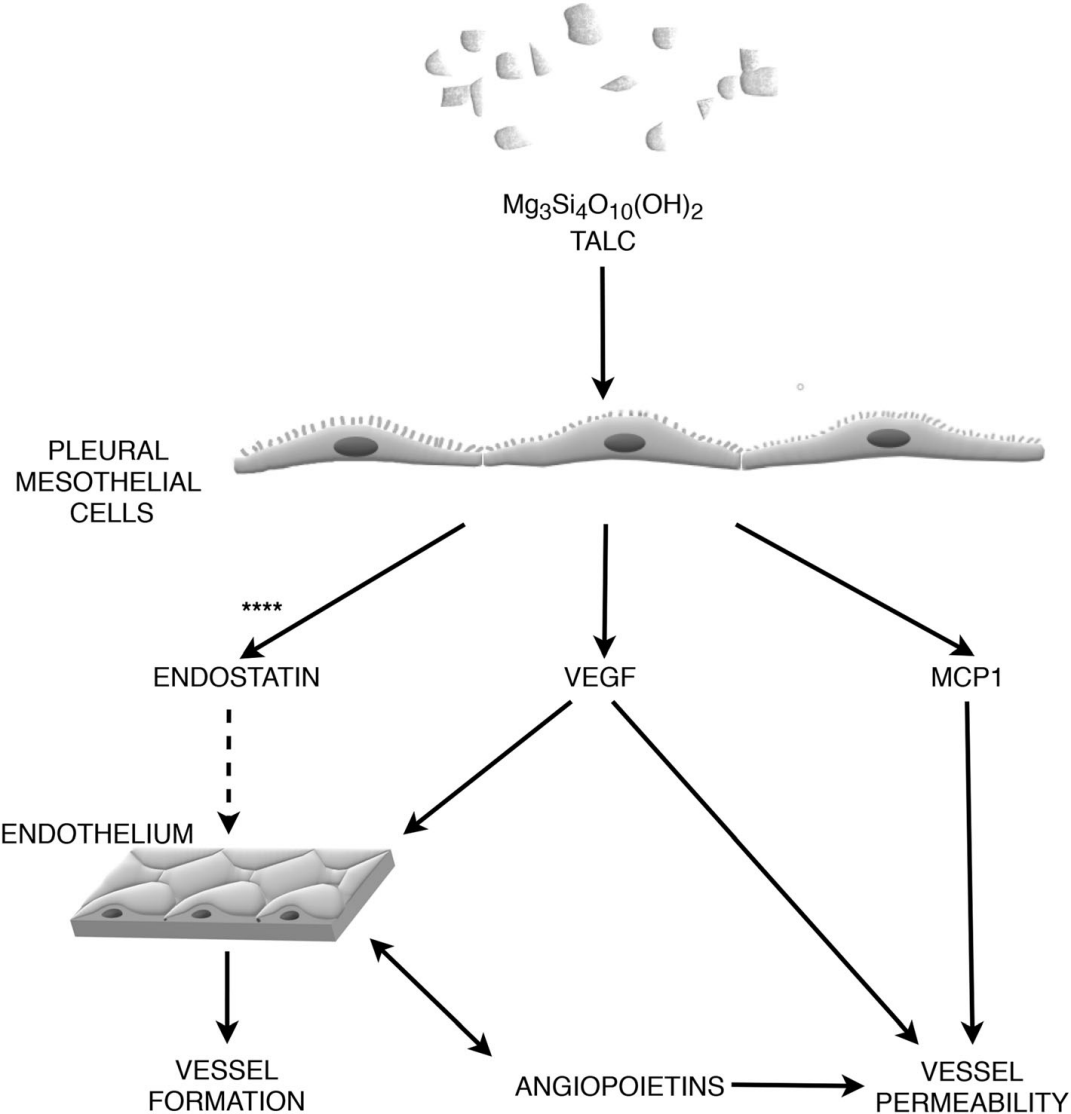
The role of angiogenesis and angiostasis in pleurodesis. In brief, pleurodesis produces not only a repair scar, but a well-vascularized and innervated connective tissue that creates a continuity between visceral and parietal pleura. The role of VEGF is ambiguous – it activates endothelial cells stimulating the formation of new vessels and induce adhesion formation but at the same time it is one of the most potent cytokines that increase vascular permeability and induce pleural fluid formation. It seems possible, that the angiogenesis-angiostasis equilibrium changes over time of the process; see text for details. Solid lines represent stimulation, dashed lines represent inhibition. **** - bidirectional and dose dependent effect on endostatin production was observed – see explanations in the text. Please, note that this is a simplified diagram. The mechanisms of angiogenesis and angiostasis involved in chemical pleurodesis are probably more complex

Angiogenesis is a process by which new capillaries are created from pre-existing vessels, while angiostasis is a negative regulatory activity of that process. Both processes play an important role in various physiological and pathological conditions, e.g. wound healing and tumor growth [103]. There is evidence that angiogenesis is involved in both pleurodesis and pleural fluid formation. This effect is mediated by endostatin, a C terminal fragment of collagen XVIII produced by its proteolytic cleavage [104] that specifically inhibits angiogenesis through disrupting endothelial cell migration, inducing cell cycle arrest and apoptosis (Fig. 4) [105].

The effect of talc administration on angiogenic balance in PMCs was evaluated by Nasreen et al. The authors used small amounts of pleural fluid collected from patients with MPE after successful talc pleurodesis. In general, the study demonstrated that talc activates endostatin release from PMCs. In fact a bidirectional and dose dependent effect was observed with higher doses showing an inhibitory effect on endostatin production. High concentration of endostatin was also found in pleural fluid collected 24 h after intrapleural talc administration. Both the conditioned media from talc activated PMC cultures and pleural fluid containing elevated level of endostatin induced apoptosis in human umbilical vein endothelial cells. These observations suggest that talc changes the angiogenesis balance in the pleural cavity towards the more angiostatic environment [106]. On the other hand, data from the rabbit model of talc pleurodesis presented by Montes et al. suggests, that pleurodesis produces not only a repair scar, but a well-vascularized and innervated connective tissue that creates a continuity between the two pleurae [107]. In this context, a local increase rather than decrease in angiogenic activity could be expected. To our knowledge, this topic was not adequately explored in human studies. Specifically, the changes over time in the pleural angiogenic/angiostatic balance after sclerosing agent administration were not addressed. It seems possible, that the equilibrium changes over time, and is different in an early and late phase of pleural reaction to sclerosants.

Another important factor involved in angiogenesis is VEGF. This inflammatory mediator is secreted by a wide range of cells present in the pleural cavity, including mesothelium, inflammatory and cancer cells [108]. Vascular endothelial growth factor is released during inflammatory pleural processes. Higher VEGF concentrations have been observed in complicated pleural effusions and pleural empyema. There are data showing that pleural thickening, low pH and glucose levels correlate with VEGF secretion into the pleural cavity [109]. There are no data on the influence of pH on VEGF production by PMCs, however there is evidence that low pH increases VEGF production in rat’s retinal extracts [110]. It has been shown that VEGF secretion is largely dependent on TGF-β. The use of anti-TGF-β antibodies blocked the production of VEGF by mesothelial cells in vivo and in vitro [111]. VEGF secretion by PMCs is also induced by bFGF [112].

The role of VEGF in pleurodesis seems ambiguous. On one hand, it activates endothelial cells stimulating the formation of new vessels and inducing adhesion formation during pleurodesis but on the other, it is one of the most potent cytokines that increase vascular permeability [113], and possibly, pleural fluid formation. Gary Lee et al. found that pleural TGF-β administration resulted in increased fluid formation compared to talc and doxycycline. The authors suggest, that this effect was probably a result of stimulation of VEGF release by TGF-β [111].

The effect of VEGF pathway blockage on recurrent pleural effusion was investigated in animal models. Two mechanisms of blockage were applied: the inhibition of VEGF receptor tyrosine kinase phosphorylation [114] and the use of anti-VEGF antibodies (bevacizumab) [115]. Both approaches decreased the permeability of local blood vessels reducing the production of pleural effusion. However, they also deteriorate the effectiveness of pleurodesis by reducing the formation of adhesions [116] and new vessels [117]. Therefore, the effectiveness of pleurodesis in patients receiving anti-VEGF antibodies in the treatment of cancer (such as colorectal cancer [118]) is likely to be reduced. These two opposite effects show that the role of angiogenesis in the pleurodesis process is complex, and so far, insufficiently understood. The therapeutic application of anti-VEGF antibodies that may be relevant for palliative treatment by reducing the production MPE will be discussed in the next chapter.

There might be an interesting link between pleurodesis and proteins from the angiopoietin group. Angiopoietins are locally produced by pleural endothelial and perivascular cells. They increase angiogenesis, vascular permeability and pleural effusion production. Kalomenidis et al. showed that angiopetin-2, but not angiopoetin-1 level, was elevated in exudative pleural effusions, and that angiopoetin-2 concentration correlated with VEGF level in pleural fluid [119]. The role of angiopoietins in pleurodesis, including inflammation and fibrosis has not been sufficiently studied.

## Potential new treatment options for MPE

An interesting concept of treatment of recurrent MPE is the local use of tissue adhesives to produce pleurodesis. Tissue adhesives are substances used for wound repair and closure of skin incisions without suture. They belong to various chemical compounds, including synthetic polymers (e.g. polycyanoacrylates), polysaccharides (e.g. chitin, chitosan) and proteins (e.g. fibrin, sercine) [120]. Data on the usefulness of these chemicals to produce pleural symphysis are still very limited. In the rat study by Cetin et al., fibrin tissue adhesive produced significantly more adhesions compared to tetracycline [121]. Importantly, this was not associated with any significant side effects. Marchi et al. found that in a rabbit model mechanical abrasion with local fibrin sealant instillation was as effective in producing pleurodesis as pleurectomy [122]. Some cases of successful pleurodesis with tissue adhesives were also reported in humans [123, 124]. However, to date, no larger studies have been performed.

Sercine is a natural protein produced by silkworms. The ability of sercine to activate fibroblasts and induce fibrosis in the pleural cavity was demonstrated in a rat study [125]. As no side effects were observed, its use seems to be a promising approach to treatment of MPE.

Although reduction of pleural fluid accumulation rate is a treatment method conceptually different than pleurodesis, this potential therapeutic option should also be mentioned. It must be admitted that the decrease of pleural fluid formation cannot be easily achieved, nevertheless, some studies showed that it is not impossible. The effect of the VEGF pathway blockage by inhibitors of VEGF receptor tyrosine kinases (PTK787) was evaluated in one experimental study by Yano et al. The authors hypothesized that the above therapeutic intervention might reduce the amount of exudate by reducing vascular permeability. Transfer of human lung adenocarcinoma (PC14PE6) cells to nude mice and subsequent treatment with different doses of PTK787 significantly reduced the incidence and volume of MPE, but did not affect the number of lung metastases. The effect on MPE was observed only with higher doses of PTK787 [126].

Recently, a significant pathobiological role of MCP-1 in MPE has been postulated. The expression of MCP-1 and its receptor CCR2 have been previously described in PMCs [127]. Thomas et al. showed that pleural fluid MCP-1 level increased significantly over time with progression of tumor stage [128]. In a study by Lansley et al. who used an animal model of benign pleurisy induced by λ-carrageenan, the blockage of MCP-1 and CCR2 resulted in a significant decrease in pleural effusion volume [129]. The same authors reported an increase of MCP-1 expression and release in both in vitro, and in vivo mouse model in response to tPA [130]. The level of MCP-1 in pleural fluid correlated positively with the fluid volume. Interestingly, blockage of the MCP-1 pathway significantly decreased the pleural fluid formation after stimulation with tPA. Therefore, the MCP-1 pathway seems to be a promising target for some new therapeutic approaches.

Pichelmayer et al. reported a reduction of MPE in one patient and reduction of malignant ascites in two patients after treatment with high doses of bevacizumab (Avastin™), a humanized VEGF neutralizing antibody [131]. Data on the blockage of other cytokine pathways are still scarce.

An indwelling pleural catheter (IPC) is increasingly used as a therapeutic option in patients with MPE and the amount of data on this treatment is rapidly growing. Although IPC was initially intended to provide an effective, long-term pleural fluid drainage, it has been shown that this treatment may eventually result in spontaneous pleurodesis. Recently, two large multicenter trials (ASAP and AMPLE-2) demonstrated a significantly higher rate of autopleurodesis in patients treated with IPC and aggressive daily pleural fluid drainage than in patients treated with IPC and every other day drainage (47 vs 24% after 12 weeks and 44.2 vs 15.9% after 6 months in ASAP and AMPLE-2 trial, respectively) [132, 133]. Also, the median time to autopleurodesis was shorter in the former group (54 vs 90 days) [132]. No significant differences in the rate of adverse events, quality of life, and patient satisfaction were found between both groups [132, 133]. Similar results, with shortened time to spontaneous pleurodesis in daily drained patients compared to those drained 2–3 times per week were recently reported by Raman et al. The use of sclerosants together with tunneled pleural catheter was associated with shorter dwell time, but higher percentage of complications [134]. The AMPLE randomized clinical trial in 146 patients showed that patients in the IPC group had a significantly shorter hospital stay than the talc pleurodesis group, however there were no significant differences in breathlessness or quality of life [135]. Olfert et al. showed that IPC is a cost-effective method when compared with talc, especially in patients with limited survival [136].

An exciting new development is the drug-eluting indwelling pleural catheter. A recent study by Bhatnagar et al. showed that the use of an IPC with a slow-release coating of silver nitrate (SNCIPC) resulted in successful pleurodesis in 8 of 9 patients with expandable lung. The median time to achieve pleurodesis was 4 days. Of the 69 recorded adverse events, 17 were device related, and there was one serious adverse event [137]. Trembley et al. demonstrated that SNCIPC used in animal model was associated with a high success rate of pleurodesis without any significant signs of silver toxicity [138]. The ongoing clinical SWIFT Trial is designed to assess whether SNCIPC enhances rates of autopleurodesis compared with uncoated IPCs [139].

New observations on pleural effusion in patients with cancer suggest that cancer cells that form pleural metastases may carry different mutations in comparison with those in the primary tumor. Han et al. demonstrated differences in EGFR and KRAS mutation status in primary and metastatic lung adenocarcinomas [140]. Agalioti et al. hypothesized that discordances may result from tumor evolution under pharmacological pressure [141]. Interestingly, in a group of patients not treated with tyrosine kinase inhibitors, 27% of metastatic tumors had EGFR mutations which had not been previously detected in primary foci [142]. In consequence, MPE therapy can potentially be personalized depending on the mutations present in the pleural metastases. Therapy could be targeted on an oncogene coming from a fusion of two genes: EML 4 (echinoderm microtubule-associated protein-like 4) and ALK (anaplastic lymphoma kinase), and inhibitors of its activity - crizotinib or ceritinib [143]. There have been reports of success in the treatment of MPE with these drugs in patients in whom other treatments have been found to be ineffective [144]. Sun et al. reported a woman with a locally advanced adenocarcinoma of the lung and a bloody pleural effusion treated with a drainage (approximately 1000 ml/day) that did not respond to chemotherapy. The amount of exudate rapidly reduced after the use of crizotinib. On the sixth day of the treatment the pleural catheter could be removed [145].

Intrapleural immune stimulation is yet the other research field currently being explored. The immune stimulants may be instilled as both anti-tumor treatment and pleurodesis inducing agents. Lansley et al. showed in a murine model that intratumoral injections of *Staphylococcus aureus* bio-product mixture induced apoptosis of mesothelioma cells through both direct and T-cell mediated mechanisms [146]. In a small study by Ren et al., patients treated with *S. aureus* superantigen mediated pleurodesis had a significantly longer survival time than that found in patients treated with talc pleurodesis (7.9 vs. 2.0 months). This difference could possibly be associated with the immunostimulating effect of *S. aureus* superantigen [147].

## Conclusions

Although pleurodesis has been used to treat patients with recurrent pleural effusion and spontaneous pneumothorax for many years, the mechanisms involved in the formation of pleural adhesions and pleural symphysis are still not fully understood. This is, at least partially, due to the complexity of the process that involves various cells as well as various mediators. Nevertheless, the majority of data show that intact mesothelial cells play a pivotal role in effective pleurodesis. It also seems that the process is largely non-specific to the type of sclerosant and involves the same ultimate pathways including activation of pleural cells, coagulation cascade, fibrin chain formation, and fibroblast proliferation. More detailed understanding of all the pathways may be a prerequisite to more effective and safer use of known sclerosants and to the development of new, perhaps partly personalized, therapeutic approaches.

## Data Availability

All data and publications discussed in the manuscript are available from the corresponding author on reasonable request.

## Abbreviations

*ALK*: Anaplastic lymphoma kinase
bFGF: Basic fibroblast growth factor
EGFR: Epithelial growth factor receptor
*EML4*: Echinoderm microtubule-associated protein-like 4
HGF: Hepatocyte growth factor
IL-1α, IL-6, IL-1β, IL8, IL-12: interleukins
IPC: indwelling pleurat catheter
MCP-1: Monocyte chemoattractant protein 1
MMPs: Matrix metalloproteinases
MPE: Malignant pleural effusion
NSAIDs: nonsteroidal anti-inflmmatory drugs
PAI 1: Plasminogen activator inhibitor-1
PDGF: Platelet derived growth factor
PE: Pleural effusion
PMCs: pleural mesothelial cells
TGF β: Transforming growth factor β
TIMP 1: Tissue inhibitor of metalloproteinase 1
TNF-α: Tumor necrosis factor α
tPA: Tissue plasminogen activator
VATS: Video-assisted thoracoscopic surgery
VEGF: Vascular endothelial growth factor
